# The effects of combined intravenous cocaine and ethanol self-administration on the behavioral and amino acid profile of young adult rats

**DOI:** 10.1371/journal.pone.0227044

**Published:** 2020-03-23

**Authors:** Alberto Marcos, Mario Moreno, Javier Orihuel, Marcos Ucha, Ana Mª de Paz, Alejandro Higuera-Matas, Roberto Capellán, Antonio L. Crego, María-Rosa Martínez-Larrañaga, Emilio Ambrosio, Arturo Anadón

**Affiliations:** 1 Psychobiology Department, School of Psychology, Universidad Nacional de Educación a Distancia, Madrid, Spain; 2 Departamento de Psicología Básica I, Facultad de Psicología, Universidad Nacional de Educación a Distancia (UNED), Madrid, Spain; 3 Departamento de Química Analítica, Química Física e Ingeniería Química, Facultad de Ciencias, Universidad de Alcalá, Ctra. Madrid-Barcelona, Alcalá de Henares, Madrid, Spain; 4 Department of Pharmacology and Toxicology, Faculty of Veterinary Medicine, Universidad Complutense de Madrid, Madrid, Spain; University of Texas at Austin, UNITED STATES

## Abstract

Under paradigms of combined intravenous cocaine and ethanol self-administration, the effects on behavior have been poorly explored. Numerous studies have found sex differences in amino acids profile and behavioral responses to each drug, yet few have focused on the interactions between cocaine and ethanol. The main objective of this work was to explore the acquisition and maintenance of intravenous self-administration behavior with a combination of cocaine and ethanol in male and female young adult rats. Likewise, the amino acids profile in blood plasma was quantified 48 hours after the last self-administration session. Male and female 52 days old Wistar rats were randomly assigned to one of 3 groups: i) saline control, ii) cocaine (1 mg/kg bodyweight/injection) and iii) cocaine and ethanol (1 mg + 133 mg/kg bodyweight/ injection). After 24 self-administration sessions carried out on a fixed-ratio-1 schedule, with a limit of 15 doses per session, 14 plasma amino acids were quantified by mean Capillary Electrophoresis technique. The curve of cocaine and ethanol combined self-administration was similar to that associated with cocaine administration alone, with females acquiring self-administration criterion before males. The self-administration of cocaine and ethanol altered the plasma concentration and relative ratios of the amino acid L-Tyrosine. In our intravenous self-administration model, females appeared more vulnerable to acquire abusive consumption of the cocaine and ethanol combination, which altered plasma L-Tyrosine levels.

## Introduction

Despite the constant appearance of new “drugs of abuse” or “recreational drugs”, and changes in their patterns of consumption, concurrent use of cocaine (an ester of benzoic acid and methylecgonine) and ethanol (an ethyl alcohol) continues to be very prevalent in many countries [1], the latter representing one of the oldest recreational drugs. The high prevalence of alcoholism or excessive ethanol consumption among cocaine users may be due to an attempt to mitigate some acute negative clinical symptoms of cocaine, such as anxiety, and/or to enhance other positive -and even desired- effects, such as euphoria or an improved perception of physical well-being [2, 3]. Like other forms of poly-consumption, the combined use of cocaine and ethanol (C+E) frequently begins during the transition period to adulthood and it is associated with important health consequences, particularly in terms of the consumption or abuse of more dangerous recreational drugs.

Although chronic exposure to the combination of C+E has already been studied in a large number of animals, intravenous self-administration has rarely been used. A model of self-administration in rats using a fixed dose of ethanol (125 mg/kg bodyweight/injection) and decreasing doses of cocaine (fading from 0.75 to 0.1 mg/kg bodyweight/injection), indicated that the cocaine dose significantly affects the total quantity of cocaine administered in each experiment but not the accumulated ethanol dose [4]. In monkeys, combined intravenous self-administration of C+E produced rates with intermediate values between cocaine and ethanol alone [5].

Metabolomics was probably the last “-omics” approach to be employed in drug addiction studies. The pioneering metabolomic studies aimed to find plasma metabolites that behave like biomarkers, both in diagnosis or to help understand the processes underlying drug addiction [6,7]. Plasma and urine samples from rats subjected to a CPP paradigm with cocaine, amphetamine and morphine have been analyzed using metabolomics techniques, demonstrating that cocaine affected four metabolites: L-threonine (L-Thr), cysteine, n-propylamine and spermidine. Plasma amino acid profiles have been proposed as biomarkers in neuropsychiatric diseases [8] like autism [9], depression [10] and epilepsy [11]. While several studies have focused on changes in plasma amino acid concentrations during acute and chronic ethanol exposure in human and animal models [12,13,14], similar studies on cocaine are scarce [15].

Using an untargeted metabolomic approach in rats, we previously investigated the effects of chronic individual and combined exposure to cocaine and/or ethanol through intravenous administration on plasma metabolic profiles. The 8 metabolites altered were mainly related to the metabolism of different amino acids, such as tryptophan, arginine, proline, methionine and cysteine [16], evidence that chronic C+E exposure could affect metabolic pathways involving different amino acids. Other studies also identified dysregulated plasma/serum amino acids or derivatives after chronic exposure to cocaine (Thr, cysteine) [17] or ethanol (alanine, tyrosine) [14,18].

Although clinical and basic research has often focused on single substances, excluding polydrug abuse patients, a less restrictive focus is now recommended that better approximates to the complexity of this issue [1,19,20]. There is therefore a need to continue developing and working on animal models that simulate polydrug abuse and in particular, the combination of C+E. Hence, this study used an animal model of poly-consumption based on intravenous self-administration in Wistar rats to assess whether the combined intravenous self-administration of cocaine (1 mg/kg bodyweight/injection) and ethanol (133 mg/kg bodyweight/injection) produces comparable behavioral effects as cocaine. In addition, we set out to study the effects of this poly-abuse on the plasma profiles of an important group of amino acids. Treatments began at an age considered to be “young adult” (52 days), and included both males and females in the experimental design to assess any possible sexually dimorphism.

## Materials and methods

### Animals and experimental design

Male and female Wistar rats were bred in-house (original supplier, Charles River Laboratories, Lyon, France), and the offspring were weaned on the 21st day and housed in single sex groups on a 12 h light/12 h dark cycle (lights on at 8.00 am), with food available *ad libitum* (A04, Scientific Animal Food, Augy, France). The procedures used had previously been approved by the Bioethics Committee of the National University for Distance Learning (UNED, Madrid, Spain) and they were performed in accordance with the EU animal welfare directive (Directive 2010/63/EU).

#### Surgical procedures

For intravenous drug administration, a polyvinyl chloride catheter (0.064" internal diameter) was introduced by surgery into the right jugular vein under isoflurane gas anesthesia: 5% induction and 2% maintenance (La Bouvet, France). Marbofloxacin (0.25 mg/kg bodyweight i.v.) was administered as a prophylactic antibiotic and buprenorphine analgesia (0.05 mg/kg bodyweight s.c.) was maintained for four days post-surgery. Catheters were flushed daily with 0.5 mL of heparin and gentamicin in order to prevent infection and to maintain patency. Twenty four hours before the first self-administration session and twenty four hours after the last session the functionality of the catheters was tested by infusing sodium thiopental (0.10 mg/kg bodyweight) and the rats that lost consciousness were considered to have a functional catheter.

#### Drug self-administration

**Fig 1:**
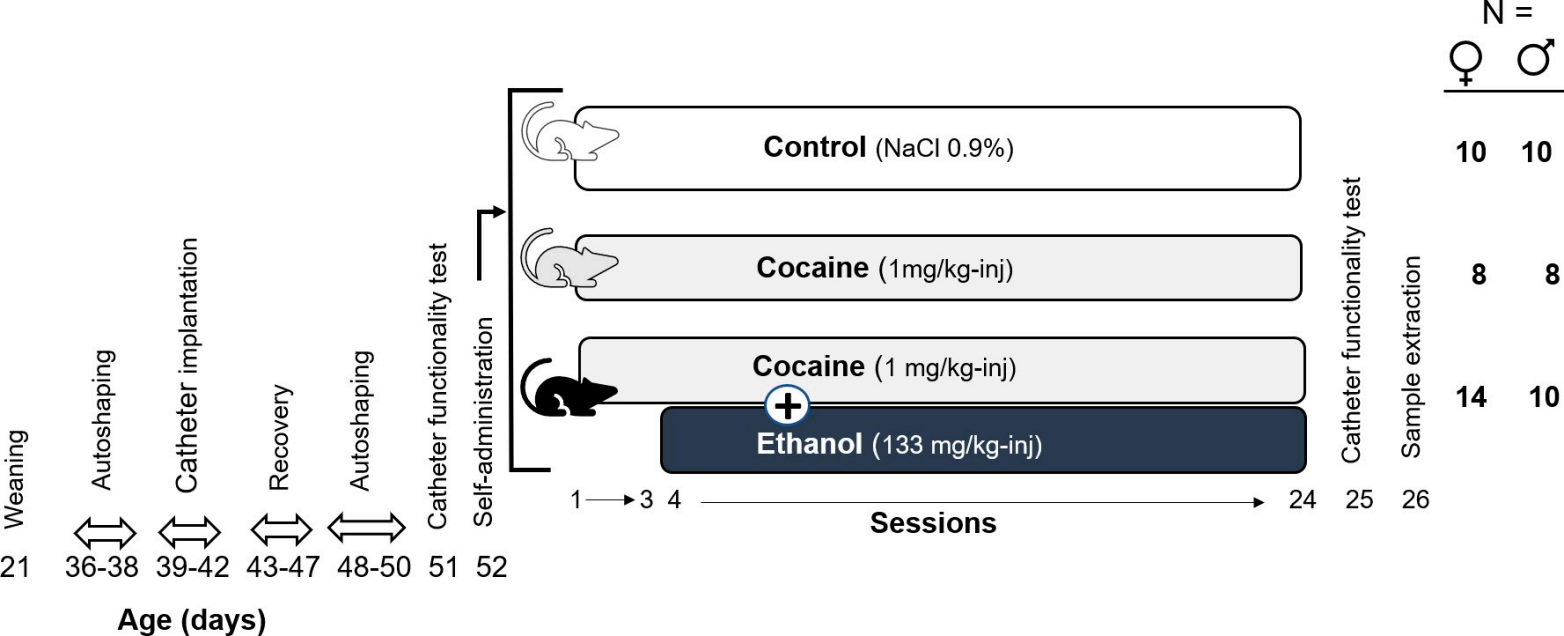
Scheme of the experimental design. From the fourth session the C+E group received ethanol along with cocaine.

Rats received seven autoshaping sessions in which were trained to self-administer food pellets under a FR-1 schedule 30 minutes sessions (4 before and 3 after catheter implantation (Fig 1). Food was not restricted during autoshaping sessions. At the age of 52 ± 1 days, the rats underwent 24 self-administration sessions (120 min, FR-1) in operant conditioning chambers (Coulbourn Instruments, Allentown, PA, USA) according to the scheme in Fig 1. Every three days the infusion pump parameters were adjusted to the weight of each animal (Harvard Apparatus, Holliston, MA, USA) and an *ad-hoc* program (Med-PC software) was used to control the effects of two levers: active (infusion of the corresponding treatment) or inactive (no effect). A light was on above the active lever to indicate the availability of the reinforcement, staying off for 20 seconds (Time-off) after each valid response or permanently when the maximum number of 15 infusions had been reached.

#### Criterion of self-administration acquisition

The sessions from one to three were considered to be an adaptation period and acquisition criterion were not considered until the incorporation of ethanol was initiated in the treatment received by the C+E rats in the fourth session. The acquisition criterion of cocaine (Coc) or C+E self-administration employed was based on that described by [21,22], adapted to our experimental design. These authors established acquisition to obtain an average administration of at least 20 mg/kg bodyweight (6 h session) of cocaine over five consecutive days. As the sessions in our experimental design last two hours, the equivalent dose would be approximately 6.7 mg/kg bodyweight/session and we rounded this up to 7 mg/kg/session. In seven or more infusions over five consecutive days, we aimed to administer: Coc, 1 mg/kg bodyweight/injection; or C+E, 1mg/kg bodyweight/injection + 133 mg/kg bodyweight/injection). Rats that took more than 12 days were considered as censored for the survival test.

### Determination of amino acids in plasma

Rats were sacrificed by decapitation and blood was collected in heparinized tubes. Plasma was obtained by centrifuging blood samples at 1500 × g (4°C) for 12 min and stored at -80°C until further analysis. Plasma samples were filtered at 30 kDa, (Centrifree, Millipore) and together with a set of standards, they were derivatized with the fluorophore NBD-F (4-fluoro-7-nitrobenzofurazan) in borate buffer (10 mM, pH 10), with L-2-aminodipidic acid as an internal standard (IS) at 200 μM. A Capillary Electrophoresis (CE) equipment PA 800 Plus (Beckman Coulter Inc.) with laser-induced fluorescence (LIF) detection was used to determine the amino acid concentrations. A fused silica capillary of 75 μm internal diameter and 50 cm effective length was used, at the settings: voltage 21 kV, laser wavelength 488 nm and 10 s sample injection at 35 mbar. Two CE-LIF methods were used in this work. A first method [23] was used to quantify ten amino acids in plasma samples using running buffer with 175 mM borate [pH 10.25] and 12 mM β-cyclodextrin (CD): L-isoleucine (L-Iso), L-leucine (L-Leu), L-ornithine (L-Orn), L-glutamine (L-Gln), L-alanine (L-Ala), L-threonine (L-Thr), glycine (Gly), L-serine (L-Ser), taurine (Tau) and L-glutamate (L-Glu). The second method involved small modifications with respect to the first, using running buffer with 80 mM borate [pH 10.25], 5% MeOH and 4 mM B-CD, and it was employed for the quantification of four other amino acids: L-Tyrosine (L-Tyr), L-phenylalanine (L-Phe), L-valine (L-Val) and L-aspartic (L-Asp). We previously optimized the separation of the peaks corresponding to these four amino acids by adjusting three variables of the running buffer: borate concentration (60, 70, 80, 90 and 100 mM), percentage of organic solvent (Methanol: 0, 5, 10, 15 and 20%) and concentration of β-cyclodextrin (2, 3, 4, 5 and 6 mM).

Resolution was calculated using the formula: 1.18 (tm2-tm1)/(wh1 + wh2), where tm1, tm2 = migration times of the peaks, and wh1, wh2 = peak widths at half-height). Linearity was established using the internal standard calibration methods from six standard solutions; and containing L-2-aminodipidic acid as IS at 200 μM. Calibration curves were established by considering the corrected peak areas (peak area to migration time ratio) both the external standard and the internal standard.

### Statistical analysis

To compare the rate of acquisition, and the percentage of males and females that reached the criterion of Coc or C+E self-administration, a survival analysis was carried out using the Kaplan-Meier method [24]. Values of p ≤ 0.05 in the Log-Rank statistic (Manel-Cox test) indicated significant differences in the acquisition curves. Those cases in which a rat had not achieved self-administration criterion in that period were considered censored cases. A mixed factorial ANOVA was used to study the self-administration sessions, response rate and response-rate/time variables. This involved an intra-subject factor of repeated measures (Session) with 24 levels that corresponded to each self-administration session, and with two factors between the groups, one of fixed effects, treatment, with three levels (saline, cocaine and C+E), and one to select the values, the sex of the rats.

In addition to each amino acid quantified and considered individually, the following variables were evaluated: branched chain amino acids (BCAAs: L-Leu + L-Iso + L-Val), aromatic amino acids (AAAs: L-Tyr + L-Phe) and the amino acid ratios (BCAA/L-Tyr), (L-Tyr/L-Phe), (L-Gln/L-Glu), (L-Ser/L-Ala) and (L-Ser/Gly). An exploratory analysis of the data was performed with the Kolmogorov-Smirnov normality test and graphical criteria, which showed slight deviations in the distributions of some of the variables studied that were corrected when necessary by transformation. The Levene statistic confirmed that in all cases the criterion of equality of variance of the distributions was met. Two-way analysis of variance was carried out (two-way ANOVA) with two factors between groups: one to select the values, the animal’s sex; and the other a fixed effects factor, the treatment group, with three levels (saline, cocaine and C+E). In the cases where a significant main effect in the treatment factor was found, planned contrasts (orthogonal) were carried out using the Helmert test, in which the first contrast compared the saline group to the rats receiving cocaine (Coc and C+E). The second contrast compared the effect of adding ethanol to cocaine (C+E against Coc). In all tests, a level of significance of p < 0.05 was considered and all the analyses were performed using the SPSS 25 statistical software.

## Results and discussion

### Acquisition

**Fig 2:**
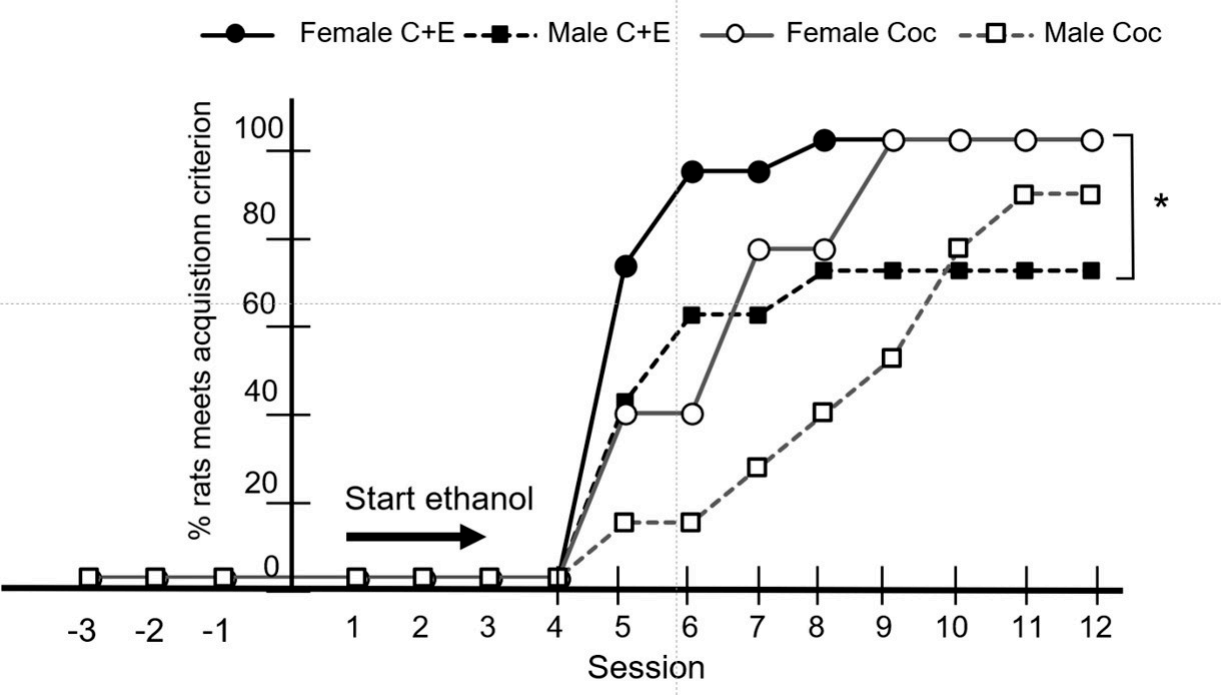
Percentage of rats that met the acquisition criterion according to treatment and sex. The asterisk shows significant differences between male and female rats administered C+E. In the first three sessions (-3 to -1) the C+E rats received cocaine alone (1 mg/kg/bodyweight/injection) and they were not assessed for the acquisition criterion. None of the rats in the saline group fulfilled the conditions of acquisition.

The percentage of female and male rats in the C+E and Coc groups that met the established acquisition was assessed in each session (Fig 2). While none of the rats administered saline met the acquisition criteria, 100% of the females met the criterion established in the first 12 days (Coc 6.8 ± 1.7, mean number of sessions ± SD and C+E 7.4 days ± 2.0), as did 70% of the males the received C+E (10.4 ± 4.9) and 87.5% of the males administered Coc (9.1 ± 2.47). Considering the sex factor stratified by treatment group (Coc and C+E), there were significant differences between males and females that self-administered C+E [χ2 (1, N = 40) = 4.94, p < 0.05]. Conversely, there were no differences between males and females in the Coc group [χ2 (1, N = 40) = 1.57, p >0 .05].

### Maintenance

**Fig 3:**
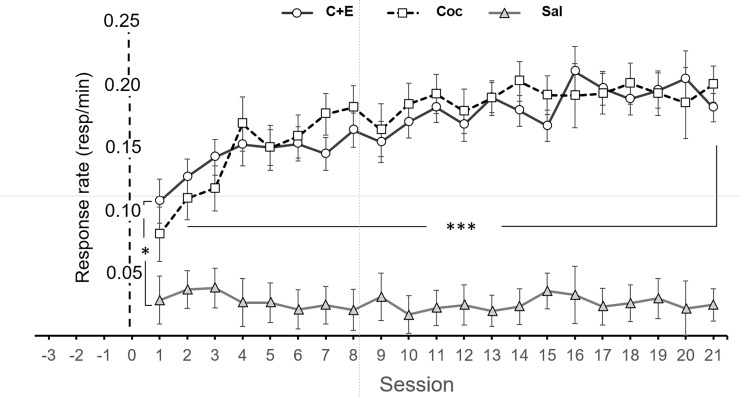
Maintenance of Coc and C+E self-administration. Mean (±SEM) of the response rate/time (lever presses per minute). The C+E and Coc rats follow a similar pattern of escalation. No session-sex interaction was detected.

In the intra-subject factor, a significant main effect of Session [F (2.92,157.87) = 6.14; p <0.001] and the Session x Treatment interaction [F (5.85,157.87) = 7.89; p < 0.001] were found. No interaction effect was observed in Session x Sex or Session x Treatment x Sex. In terms of the simple effects of the significant interaction, the differences were found from session 4 [F (2,54) = 6.78; p < 0.05]. In this session, the group administered saline differed from the C+E group (p < 0.05), with a higher response rate in the group administering both drugs relative to those that received saline, with no significant differences between the Coc and the saline or the Coc and C+E groups. From day 5, the simple effects interaction highlighted significant differences [F (2.54) = 9.92; p < 0.001] between the saline and Coc rats (p < 0.05) and the saline and C+E rats (p < 0.001), with a significantly higher response rate in the animals that received the drug(s) than in the saline group, yet there were no differences between the Coc and C+E rats. These differences persisted until the end of the behavioral testing (Fig 3).

Regarding the intra-group factors, a main effect of Treatment was found [F (46.67,157.87) = 46.67; p < 0.001]. The orthogonal Helmert contrasts for this factor highlighted significant differences between the saline control group and the groups that self-administered the drugs when considered jointly (saline vs. Coc and C+E; p <0.001) but not between the Coc and C+E groups (p >0 .05). Gabriel post hoc tests confirmed these results (saline vs. cocaine p < 0.001, saline vs. C+E p < 0.001, cocaine vs. C+E p > 0.05). These effects were the same regardless of the sex of the rats, since no significant main effect of Sex or a Sex x Treatment interaction were found.

### Optimization of L-Tyr, L-Phe, L-Val and L-Asp assessment

**Fig 4:**
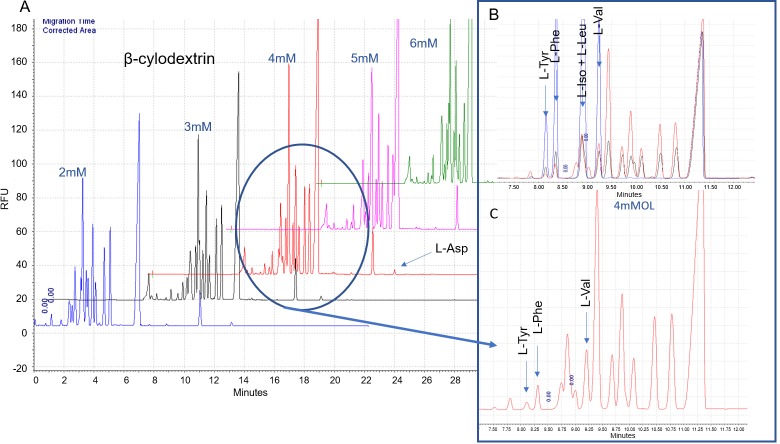
Optimization of L-Tyr, L-Phe, L-Val and L-Asp assessment. (A) Electropherograms of the optimization of the concentration of β-CD in running buffer. (B) Detail of the separation achieved with 4 mM β-CD: (C) Superposition of electropherograms: red, plasma sample; black, mix of 21 amino acids (100 mM) and blue, L-Tyr, L-Phe, L-Iso, L-Leu and L-Val. (0.5 mM).

The best running buffer conditions for the separation of the peaks corresponding to L-Tyr, L-Phe, L-Val and L-Asp were: 80 mM borate at pH 10.25, 5% MeOH and 4 mM B-CD (Fig 4). The resolution between peaks, was equal or higher than 2. A mix of 21 amino acids [L-lysine (L-Lys), L-methionine (L-Met), L-proline (L-Pro), L-histidine (L-His), L-arginine (L-Arg), L-asparagine (L-Asn), L-cysteine (L-Cys), L-Iso, L-Leu, L-Orn, L-Gln, L-Ala, L-Tre, Gly, L-Ser, Tau, L-Glu, L-Tyr, L-Phe, L-Val and L-Asp] at 100 μM concentration was used to a selectivity test. As shown in Fig 4C, there is no effect of the sample matrix (plasma) on the separation of the different amino acids, no alteration in migration, peak width or selectivity is observed.

The calibration curves, obtained as the ratio corrected-area-AA/corrected-area-IS vs concentration-AA, were linear in the range of concentration tested L-Phe (R^2^ = 99.8) and L-Tyr (R^2^ = 99.2) between 30 and 370 μM/L, L-Val (R^2^ = 99.8) between 60 and 600 μM/L and L-Asp (R^2^ = 99.9) between 3 and 82,4 μM/L. The LOD, evaluated as S/N = 3, was 0,35 μMl/L for L-Asp y L-Val, 0,45 μMl/L for L- L-Phe and 0,7 for L-Tyr, while the LOQ, evaluated as S/N = 10, was 1,1 μMl/L for L-Asp y L-Val, 1,35 μMl/L for L- L-Phe and 2,2 for L-Tyr.

### Tyrosine

**Fig 5:**
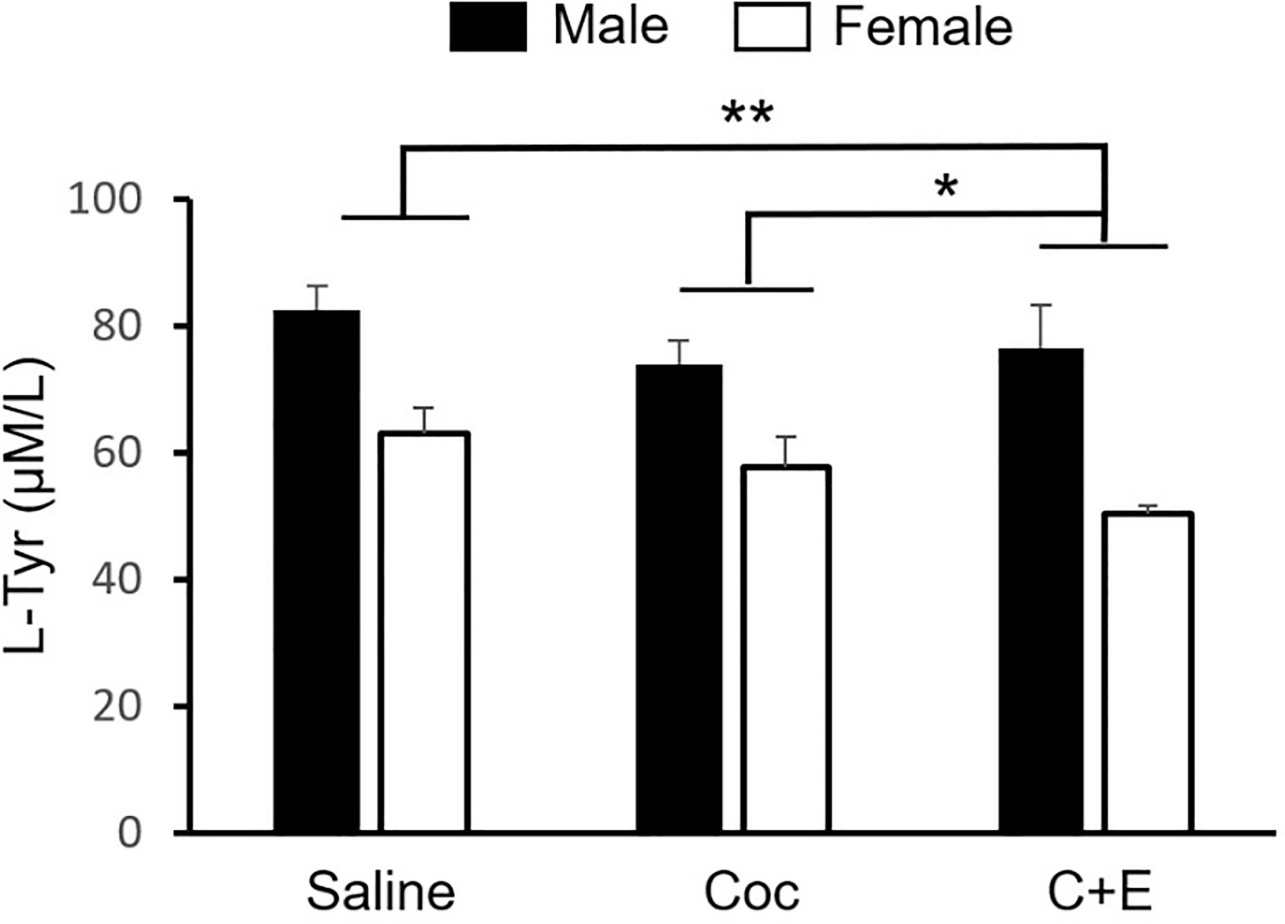
Plasma concentration of L-Tyr according treatment and sex. Mean (±SEM) of L-Tyr. Effects of Sex, reflected by the higher concentration in male rats, and Treatment were found (C+E vs. Coc; C+E vs. Sal). * P<0.05; ** P<0.001.

Regarding tyrosine (L-Tyr), there was a significant effect of Sex [F (1,54) = 58.90; p <0 .001], which was stronger in males (M = 77.84, SD = 16.76) than in females (M = 50.06, SD = 9.71) (Table 1), and there was also a main effect of treatment [F (2,54) = 7.32; p < 0.01]. The post hoc analysis showed significant differences between the C+E and Saline group (p < 0.001), and between the C+E and Coc rats (p < 0.05), with a lower concentration (μM/L) in the C+E group (M = 61.11, SD = 20.29) relative to the Coc (M = 65.70, SD = 11.70) and Saline animals (M = 72.78, SD = 15.53). There were no significant differences between the Saline and Coc treatment groups (Fig 5).

**Table 1:** Effect of self-administration of cocaine and C+E on plasma amino acid concentrations (μM/L).

	Saline			COC			C+E		
	Total	Males	Females	Total	Males	Females	Total	Males	Females
L-Leu	185.95	207.64	164.25	173.59	179.52	167.66	179.86	175.43	183.03
±SD	42.81	50.55	16.38	22.22	19.09	24.76	25.93	19.55	29.99
L-Iso	105.57	110.84	100.31	106.71	113.23	100.19	102.48	102.39	102.55
±SD	18.21	20.74	14.44	16.03	16.76	13.13	14.11	13.73	14.89
L-Orn*	74.33	83.10	65.56	72.68	82.64	62.72	75.89	91.97	64.41
±SD	14.38	13.54	9.06	16.94	16.47	10.80	20.05	16.26	13.70
L-Gln*	766.83	739.19	794.47	691.84	636.61	747.08	714.56	672.09	744.89
±SD	117.27	111.18	122.38	94.50	45.18	100.61	102.35	87.25	104.34
L-Ala*	472.93	523.31	422.55	474.48	495.68	453.27	475.82	519.02	444.96
±SD	75.53	68.35	41.61	47.20	37.42	48.44	72.36	78.24	50.65
L-Thr	330.12	348.77	311.47	291.16	295.30	287.01	313.56	309.17	316.69
±SD	70.37	69.72	69.43	54.52	57.54	54.95	62.52	60.89	65.75
Gly*	395.26	448.62	341.91	369.47	378.94	359.99	370.93	434.56	325.49
±SD	86.43	81.77	52.51	78.78	72.02	88.93	89.05	84.59	61.13
L-Ser	323.65	348.50	298.79	308.44	302.37	314.51	308.78	313.02	305.75
±SD	49.06	55.65	24.75	59.23	51.24	69.35	55.00	63.12	50.69
Tau	312.11	340.76	283.46	317.52	247.61	387.43	277.47	263.11	287.73
±SD	124.01	153.29	84.52	212.97	63.93	286.24	61.27	74.29	50.50
L-Glu	127.51	140.25	114.77	119.20	111.19	127.21	128.00	139.07	120.10
±SD	29.50	30.50	23.39	41.35	20.82	55.53	19.89	14.84	19.64
L-Tyr*#	72.78	82.59	62.98	65.70	73.75	57.65	61.11	76.37	50.22
±SD	15.53	11.48	12.78	11.70	11.01	4.91	20.29	23.97	4.84
L-Phe*	65.66	68.47	62.86	64.51	67.72	61.30	65.21	71.41	60.79
±SD	7.85	9.04	5.55	6.80	7.33	4.69	8.63	6.06	7.47
LVal*	210.06	219.19	200.93	210.91	222.99	198.84	211.03	219.27	205.15
±SD	25.80	25.17	24.22	25.27	25.25	19.94	24.13	22.61	24.22
L-asp	24.82	27.77	21.88	24.96	19.84	30.08	22.56	24.03	21.51
±SD	10.96	13.66	6.91	14.96	4.81	19.91	6.59	6.15	6.91

*Asterisks indicate significant Sex differences

# Indicate significant effects of Treatment

### BCAA to Tyr molar ratio

**Fig 6:**
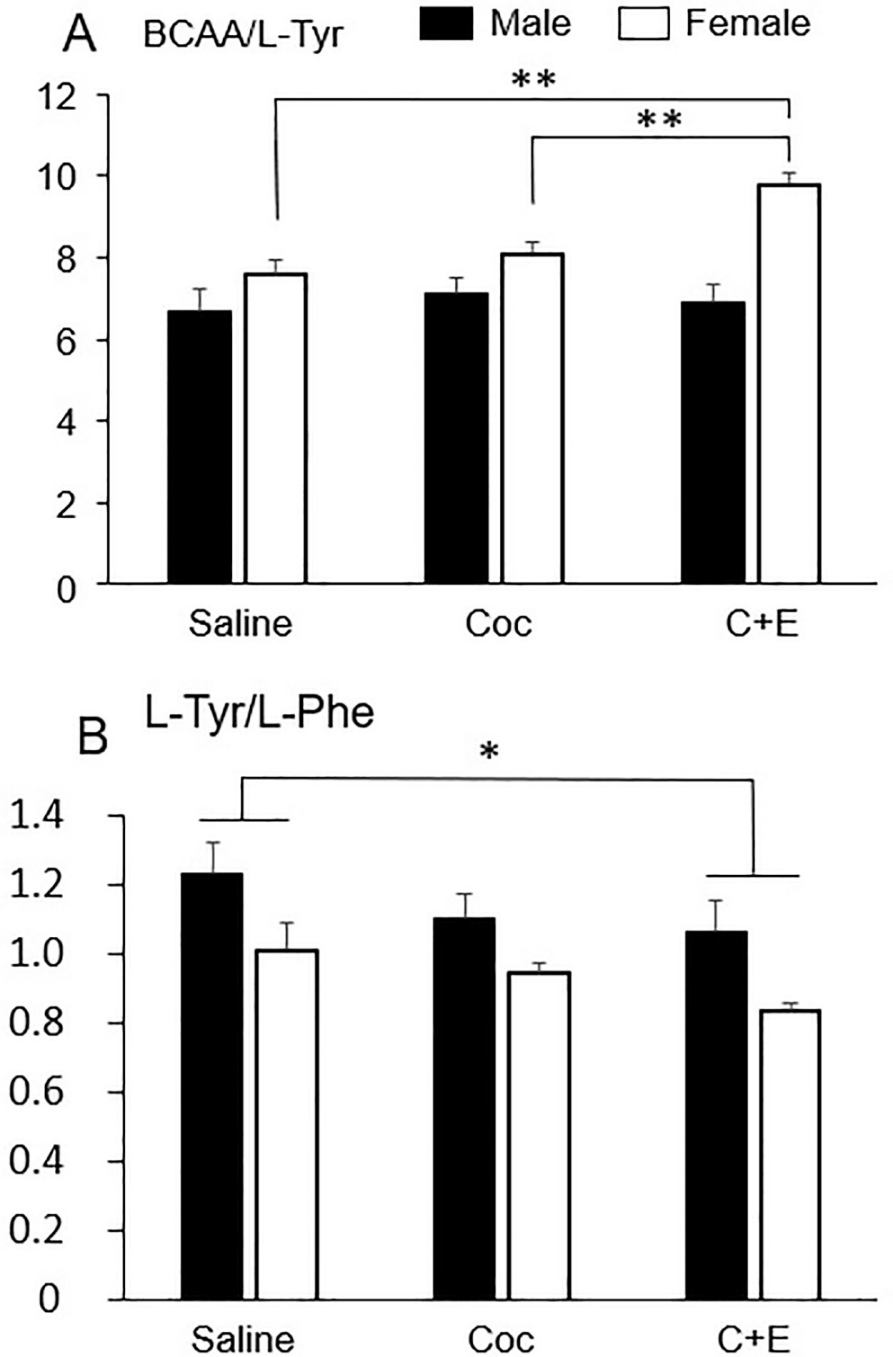
Effects of cocaine and C+E on amino acids molar ratios. (A) Mean (±SEM) of the BCAA/L-Tyr molar ratio. Effects by Sex, Treatment and their interaction were significant. The ratio was higher in female Coc rats and even higher in those that received C+E. (B) Mean (±SEM) of the L-Tyr/L-Phe molar ratio, where a Treatment effect (C+E vs. Sal) was found. Sex differences were also seen, with a smaller ratio in females with Coc and even lower with C+E compared to the males. * p<0.05; ** p<0.001.

An ANOVA analysis identified significant differences in the BCAA to tyrosine molar ratio (BCAA/L-Tyr) for the Sex factor [F (1.54) = 21.68; p < 0.001], with higher values in females (M = 8.67, SD = 1.44) than in males (M = 6.88, SD = 1.49), and an effect of Treatment [F (2.54) = 4.80; p < 0.05]. Post hoc tests reflected a higher ratio in the C+E group compared to the Saline group (p < .01), and a significant interaction of Sex x Treatment [F (2,54) = 4.11; p < 0.05] was also found. In the comparisons of the means by pairs to study the simple effects (Bonferroni correction), significant differences were found between the different levels of the Treatment variable in the female groups [F (2,54) = 9.60; p < 0.001]. Specifically, there were significant differences between the C+E and Saline group (p < .001), and between the C+E and Coc rats (p < 0.05). This difference was not evident in males (Fig 6) (Table 2).

**Table 2:** Effect of self-administration of cocaine and C+E on plasma amino acid molar ratios.

	Saline			COC			C+E		
	Total	Male	Female	Total	Males	Females	Total	Males	Females
SUM AA	3467.58	3688.99	3246.17	3291.17	3227.40	3354.93	3307.26	3410.89	3233.24
±SD	461.47	507.44	288.33	470.51	301.93	611.50	327.85	340.06	309.75
AAA*	138.44	151.06	125.83	130.21	141.47	118.95	126.33	147.78	111.00
±SD	17.73	11.63	13.22	15.49	12.79	7.80	26.41	26.94	11.17
BCAA*	501.58	537.66	465.49	491.21	515.74	466.68	493.37	497.09	490.72
±SD	79.89	88.96	51.64	60.12	58.97	53.77	55.46	45.60	63.11
BCAA/L-Tyr&*#	7.13	6.70	7.57	7.60	7.10	8.10	8.58	6.89	9.79
±SD	1.53	1.77	1.17	1.05	1.12	0.73	1.95	1.57	1.11
L-Tyr/L-Phe*#	1.12	1.23	1.01	1.02	1.10	0.94	0.93	1.07	0.83
±SD	0.28	0.28	0.25	0.17	0.21	0.09	0.23	0.30	0.09
L-Gln/L-Glu*	6.28	5.47	7.10	6.18	5.86	6.50	5.73	4.87	6.35
±SD	1.56	1.25	1.43	1.56	0.88	2.05	1.35	0.73	1.37
L-Ser/L-Ala*	0.69	0.68	0.71	0.65	0.61	0.69	0.66	0.61	0.69
±SD	0.11	0.15	0.05	0.12	0.10	0.13	0.13	0.13	0.11
L-Ser/L-Gly*	0.84	0.79	0.89	0.85	0.81	0.89	0.86	0.74	0.96
±SD	0.13	0.13	0.12	0.15	0.12	0.18	0.20	0.18	0.16

*Asterisks indicate significant Sex differences

# Indicate significant effects of Treatment

& Sex x Treatment interaction effects.

**SUM AA:** sum of fourteen amino acids (μM/L); **AAA:** aromatic amino acids (L-Tyr+L-Phe) (μM/L); **BCAA:** branched chain amino acids (L-Iso+L-Tyr+L-Phe) (μM/L).

### Tyrosine to phenylalanine molar ratio

The tyrosine to phenylalanine molar ratio (L-Tyr/L-Phe) showed a significant main effect of Sex [F (1,54) = 16.56; p < 0.001], with a higher average in males than in females (M = 1.14, SD = 0.07 vs. M = 0.92, SD = 0.17), and a significant Treatment effect [F (2.54) = 4.97; p < 0.05]. The post hoc Gabriel test indicated that the differences between the Saline control and the drug treatment groups were stronger in the C+E group (p < 0.01), with no significant differences between the Coc and Saline rats, or between Coc and C+E rats. The lowest ratio corresponded to the C+E group (M = 0.93 SD = 0.23; see Fig 6 and Table 2). In the proposed contrasts (Helmet method), significant differences in the L-Tyr/L-Phe ratio were found between the Saline group and the Coc and C+E groups together (p < 0.05), with no significant differences between the Coc and C+E groups.

### Global discussion

The aim of this study was to establish a procedure that allows the effect of combined intravenous C+E self-administration to be assessed in laboratory rats. Given the high frequency of the concurrent consumption of both these drugs of abuse in humans and the strong ecological validity of the animal model, having an operant conditioning procedure that readily enables both drugs to be chronically self-administered together in young animals of both sexes offers new perspectives to study the consequences of this type of poly-consumption in humans. Here, plasma samples were obtained from the animals to subsequently determine the levels of the different types of amino acids, considering that alterations to the concentrations of certain amino acids may play a role in diseases like autism [9] and depression [10].

Intravenous self-administration of ethanol in rats is not particularly widespread, the low levels of cumulative ethanol intake probably due to its limited pharmacokinetic scope [25,26,27]. In addition, there appear to be few studies in which animals were self-administered both cocaine and ethanol together. In one of these, adult male rats self-administered cocaine and ethanol as a procedure for intravenous pre-exposure to ethanol, to later study intravenous self-administration of ethanol alone in these animals [4]. The other study addressed whether self-administration of ethanol in combination with another drug, such as gamma hydroxybutyrate acid (GHB), flunitrazepam or cocaine increased the reinforcing properties of these drugs in rhesus monkeys (three males and three females: [5]. All the rhesus monkeys used in this study had a previous history of other drug consumption and were not experimentally “naïve”. Unlike these two studies, the present study attempted to obtain a more detailed definition of combined cocaine and ethanol self-administration behavior in order to define a method that may be useful for other neurobiological studies. As such, the acquisition and maintenance phases of this behavior in young rats of both sexes that have not previously been exposed to any drug of abuse was analyzed, comparing this with the self-administration of cocaine alone. The results indicate that the combination of cocaine and ethanol facilitates swifter learning of self-administration behavior in females relative to males, a difference that was not found between sexes when cocaine was the only drug self-administered. These results suggest that males and females may have different sensitivities to the reinforcing properties of the combination of both drugs, with female rats apparently more susceptible to these properties in the initial acquisition phase of this behavior. Nevertheless, once this behavior is acquired and during the subsequent maintenance phase, no differences were found between females and males in the C+E group or with those that self-administered cocaine only. However, the difference between the rats that self-administered a drug and those of saline serum is clearly significant, demonstrating that the C+E combination is a clear reinforcing stimulant for animals of both sexes, and that the presence of ethanol does not diminish cocaine’s reinforcing properties, at least in laboratory rodents and in the specific conditions of this study.

It must be noted that the results of this study do not concur with those from rhesus monkeys [5], in which self-administration of both drugs in combination (ethanol 50, 100 or 200 mg/kg bodyweight/injection; cocaine 0.01 or 0.03 mg/kg/injection) reduced cocaine’s reinforcing properties. However, it is possible that the difference in these results is not just due to the species used but also, to the considerably lower doses of cocaine administered to the monkeys (0.01 to 0.03 mg/kg/injection) relative to those used here in rats (1 mg/kg bodyweight/injection). Indeed, at a constant concentration of ethanol (125 mg/kg bodyweight/injection) similar to that used here (133 mg/kg bodyweight/injection), the concentration of cocaine was reduced progressively from 0.75 to 0.1 mg/kg bodyweight/injection, and the response to C+E self-administration from the dose of 0.40 mg/kg bodyweight/injection cocaine (125 mg/kg bodyweight/injection of ethanol plus 0.4 mg/kg bodyweight/injection of cocaine) was weaker than that of cocaine alone (0.4 mg/kg/injection: [4]. Hence, a minimum dose of cocaine may be required for a given dose of ethanol to maintain the self-administration behavior of both drugs in combination. Alternatively, the importance of the formation of the metabolite cocaethylene in maintaining the C+E self-administration behavior cannot be ignored. Ethanol inhibits the hydrolysis of cocaine’s methyl ester group by carboxylesterases, reducing the production of the non-psychoactive metabolite benzoylecgonine, which means that cocaine requires more time to be metabolized and it is in the plasma for longer. Conversely, these carboxylesterases catalyze the shift of cocaine to cocaethylene [28,29,30]. Cocaethylene enhances motor activity and it helps to establish instrumental behavioral patterns [31], displaying similar reward effects to cocaine when assessed in conditioned place preference (CPP) and self-administration paradigms [31,32]. C+E act synergistically to increase in the mesolimbic reward pathways [33]. The release of dopamine in the nucleus accumbens (NAcc) is known to be enhanced by ethanol [34,35] and there is more extracellular dopamine in the NAcc of rats treated with C+E than in those treated with cocaine alone [36].

After quantifying the plasma amino acid concentrations, significant differences were detected between sexes for seven of the 14 amino acids studied (L-Orn, L-Gln, L-Ala, Glycine, L-Tyr, L-Phe and L-Val), their levels generally higher in males than in females (except for L-Gln) regardless of the treatment. There were also differences between sexes for the AAAs and BCAAs, and in the L-Gln/L-Glu ratio. It is notable that the tyrosine concentrations differed depending on the treatment and relative to the control animals, there was less of this amino acid in males and females of both the Coc and C+E groups. Furthermore, significant differences in the L-Tyr/L-Phe and the BCAA/L-Tyr ratios were identified in the treatment groups. It is widely known that L-L-Phe and L-Tyr are precursors of catecholamines (Dopamine, norepinephrine and epinephrine). Phenylalanine is an essential amino acid and its conversion to tyrosine is catalyzed by phenylalanine hydroxylase, whereas tyrosine hydroxylase (TH) participates in the conversion of tyrosine to L-DOPA, a precursor of dopamine. Although hydroxylation of phenylalanine to tyrosine mainly occurs in the liver, it also takes place in the CNS and the adrenal medulla where it is catalyzed by TH [37]. The diminished basal levels of L-Tyrosine that were found in female rats could be related to sex differences in the functionality of the adrenal glands. Thus, it is well known that the adrenal medulla is the main organ where the conversion of L-Tyr into cathecholamines take place. Recently, it has been described that the functionality of adrenal gland is higher in female than male mice [38]. A greater conversion rate of L-Tyr into catecholamines in the female rats of our study could explain the low levels of this amino acid in the blood stream of these animals.

The BCAA/L-Tyr ratio is known to be a good indicator of liver metabolism, meaning it may also be useful as a marker for the possible alterations in the relationship between BCAAs and tyrosine in consumers of cocaine and ethanol. Likewise, L-Tyr/Phe ratio is particularly low in problematic cocaine consumers [15] and in individuals with Parkinson’s disease [39]. As both pathologies have less dopamine in the brain, this may reflect the changes in the dopamine amino acid precursor, tyrosine, and the reduction in this amino acid associated with cocaine self-administration, as seen here, may be a possible indicator of altered dopamine levels in the brain. In the synthesis of catecholamines, tyrosine hydroxylase is the rate-limiting enzyme for dopamine synthesis. Given that in our study there is a clear sexodimorphism in female lower tyrosine levels, it could be considered that estrogens can have a role in our results because it has been demonstrate that regulate the expression of TH [40]. In this sense, in adult rodents, mice and rats, ovariectomy reduces tyrosine hydroxilase inmunoreactive cells number in VTA and replacement with β-oestradiol agonist reduced or prevent cell loss [41].

## Conclusions

In summary, this study shows that young laboratory rodents of both sexes will readily self-administer cocaine and ethanol in combination. The advantages that intravenous self-administration offers, both in handling behavioral variables and in controlling pharmacokinetics, means this approach should be considered in poly-consumption studies of these two substances. Differences between sexes were observed in the acquisition phase and changes in the metabolic markers related to tyrosine were noted. Other studies of distinct behavioral and neurophysiological patterns will be required to understand the underlying processes in this prevalent form of poly-consumption.
